# Effect of Synthetic Peptides Identified in the Bullfrog Skin on Inflammation and Oxidative Stress Control: An In Vitro Analysis

**DOI:** 10.3390/molecules30102223

**Published:** 2025-05-20

**Authors:** Silvânia Mól Pelinsari, Patricia da Silva Mattosinhos, Manoela Maciel dos Santos Dias, Rosinéa Aparecida de Paula, Romulo Dias Novaes, Emerson Ferreira Vilela, Giuseppe Valacchi, Reggiani Vilela Gonçalves

**Affiliations:** 1 Department of General Biology, Federal University of Viçosa, Viçosa 36570-900, MG, Brazil; silvania.pelinsari@ufv.br (S.M.P.); patricia.mattosinhos@ufv.br (P.d.S.M.); 2 Department of Animal Biology, Federal University of Viçosa, Viçosa 36570-900, MG, Brazil; manoelamsdias@gmail.com (M.M.d.S.D.); neia-depaula@yahoo.com.br (R.A.d.P.); 3 Department of Structural Biology, Biomedical Science Institute, Federal University of Alfenas, Alfenas 37130-000, MG, Brazil; romuonovaes@yahoo.com.br; 4 Minas Gerais Agricultural Research Agency (EPAMIG Sul), Experimental Field of São Sebastião do Paraíso, São Sebastião do Paraíso 37959-899, MG, Brazil; emerson.vilela@epamig.br; 5 Department of Animal Science, Plants for Human Health Institute, North Carolina State University, Kannapolis, NC 28081, USA; gvalacc@ncsu.edu; 6 Department of Environmental and Prevention Sciences, University of Ferrara, 44121 Ferrara, Italy; 7 Department of Food and Nutrition, Kyung Hee University, Seoul 02447, Republic of Korea

**Keywords:** peptides, inflammatory response, skin diseases, bullfrog, cytokines

## Abstract

(1) Background: This study evaluated the potential of a synthetic peptide (SGHPGAMGPVGPR), identified in the bullfrog (*Lithobates catesbeianus*) skin, in regulating inflammation and oxidative stress using RAW 264.7 macrophages; (2) Methods: Molecular docking determined its optimal interaction with cyclooxygenase (COX-2) an enzyme related to the production of prostaglandins, which play a crucial essential role in the inflammatory response. The peptide was commercially synthesized company, and its antioxidant capacity was assessed using DPPH and FRAP assays. Cell viability, nitric oxide (NO) levels, catalase (CAT), superoxide dismutase (SOD) and glutathione s-transferase (GST) activity, interleukin-6 (IL-6) and tumor necrosis factor-alpha (TNF-α) gene expression and cell production were additionally quantified. (3) Results: The peptide SGHPGAMGPVGPR, designated as P1, exhibited remarkable free radical scavenging capacity, antioxidant, and anti-inflammatory activities. No significant difference was observed in SOD and CAT activity in P1-treated macrophages, likely due to downregulation in the Nrf2/HO-1 pathway. Reduced GST activity was observed in these cells, which was potentially associated with TNF-α downregulation; (4) Conclusions: These findings suggest that P1 modulates the antioxidant response through pathways independent of classical antioxidant enzymes. Furthermore, decreased IL-6, COX2, and nuclear factor kappa B (NF-κB) expression was observed, indicating the involvement of a key pathway in the regulation of the OxInflammation process.

## 1. Introduction

Inflammation is a collective immune response of the body against certain stimuli, such as microbial infections, oxidative stress, and tissue or cell damage [1]. However, when deregulated, it can cause tissue damage and contribute to the development of acute and chronic inflammatory diseases. Several signaling pathways are involved in immune responses (e.g., NF-κB), upregulating pro-inflammatory mediators such as cytokines (e.g., tumor necrosis factor-alpha—TNF-α and IL-6), chemokines (e.g., monocyte chemoattractant protein-1—MCP1 and macrophage inflammatory proteins—MIP), and enzymes (e.g., COX) [2]. These mediators coordinate the immune response and promote the recruitment of additional cells to the inflammation site [3].

Inflammation and oxidative stress are interconnected, with inflammation playing a critical role in innate immune responses and significantly contributing to the development of oxidative stress [4]. Oxidative stress, resulting from the imbalance between reactive oxygen species (ROS) production and antioxidant defenses, to neutralize these compounds, culminate in bimolecular damage (e.g., lipid, protein and nucleic acids oxidation) [5]. This process is associated with OxInflammation, which involves an increased release of free radicals and reactive oxygen species (ROS) that act as secondary messengers and promote the release of inflammatory markers, creating a positive feedback loop between oxidative stress and inflammation [6]. This concept emerged from the observation that, in chronic inflammation, the increase in ROS is not only a marker of stress but also a mediator in the amplification of inflammation.

The OxInflammation process is intensified by inflammatory pathways such as toll-like receptors (TLRs), which detect damage-associated molecular patterns (DAMPs) [7]. In macrophages, dendritic, and mast cells, TLRs detect excessive activation triggering pro-inflammatory mediators (e.g., cytokines and chemokines) production, which coordinate the immune response and promote leukocyte recruitment to the inflammation site [8]. It has also been demonstrated that OxInflammation is associated with structural modifications of proteins, lipids, and nucleic acids, changing the function of several OxInflammatory modulators, including redox-sensitive transcription factor signaling NF-κB and COX-2 [9,10]. Hence, it may even contribute to chronic inflammation or cell death and affect the innate immune system [11]. For these reasons, there is a real need to develop effective therapies to control OxInflammation inside cells, especially macrophages. Animal peptides stand out as a promising therapeutic approach, since they exhibit antioxidant and anti-inflammatory effects through interactions with intracellular components and membrane receptors [12,13]. Current evidence indicates that animal peptides can protect cells against oxidative stress by preventing excessive ROS production and lipid peroxidation [14,15]. Moreover, recent studies indicate that animal peptides support the maintenance of proteostasis under oxidative conditions by regulating molecular signaling involved in inflammation, degeneration, necrosis, and apoptosis [16,17]. In addition, animal peptides can modify cell viability by altering mitochondrial membrane permeability and calcium metabolism, as well as the level of apoptosis regulatory proteins such as B-cell lymphoma 2 (Bcl-2) and Bcl-2-associated X protein (Bax) [18,19].

In recent years, significant advancements in peptide therapies have led to extensive research on peptides that act on multiple receptors. Many of these peptides have been approved by regulatory agencies to prevent and/or treat diseases such as dermatitis, psoriasis, and vitiligo [20]. Animals’ skin (e.g., amphibians and reptiles) is a natural source of active peptides with a broad spectrum of biological functions. Probably because they are continuously exposed to environmental challenges (e.g., pathogens, predators, chemicals, and radiation), animal skin has developed a complex chemical defense system, including antioxidant (AOPs) and anti-inflammatory (AIPs) peptides as a protective skin response [21]. For example, the peptide-I (TWYFITPYIPDK), isolated from the tropical frog *Physalaemus nattereri*, exhibits antioxidant activity. At the same time, the peptide salamandrine-I (FAVWGCADYRGY-NH2), derived from the skin secretions of *Salamandra salamandra*, shows the ability to scavenge free radicals [22].

The application of amphibian-derived peptides for wound healing is being investigated to identify potential additional benefits. Accordingly, exploring the relevance of these peptides in the treatment inflammatory skin diseases is a rational path to pursue. Methods such as Enzyme-Linked Immunosorbent Assay (ELISA), gene expression studies by polymerase chain reaction (PCR), and oxidative stress analyses in cell cultures are essential to understand their biological actions and reveal the role of animal-derived peptides and their potential applications in inflammatory diseases. Previous findings presented by our research group showed that the bullfrog skin (*Lithobates catesbeianus*), increased fibroblast proliferation and a reduced the inflammatory infiltrate in a murine model of skin wound healing [14]. In addition, TRL-4 and COX-2 were downregulated, culminating in reduced IL-6 and interleukin-1β (IL-1β) levels. In this previous study, 71 peptide sequences were identified in the skin fraction digested with trypsin [14]. Among them, the peptide SGHPGAMGPVGPR showed the best results in molecular docking assays, indicating greater affinity with COX-2, a central pro-inflammatory modulator. Accordingly, this relevant peptide is being further investigated for its immunomodulatory potential in regulating oxidative stress and inflammatory response in RAW 264.7 macrophage cells.

## 2. Results

### 2.1. Peptide

**Figure 1:**
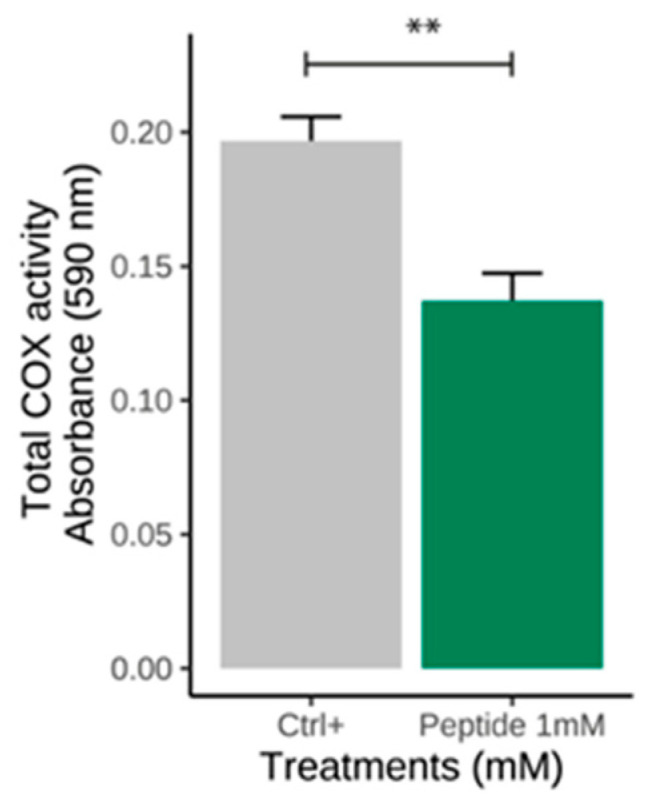
Effect of the peptide SGHPGAMGPVGPR (P1) on total COX activity. RAW 264.7 cells were subcultured at a density of 1 × 10^8^ cells/mL and stimulated with LPS at 1 μg/mL for 4 h. Cells were collected by centrifugation at 2000 rpm for 10 min at 4 °C and were resuspended in DMEM. The cells were then treated with the peptides, and the ELISA kit was performed. Significant by *t*-test (** *p* ≤ 0.01). CTRL: control (cells stimulated with LPS).

**Figure 2:**
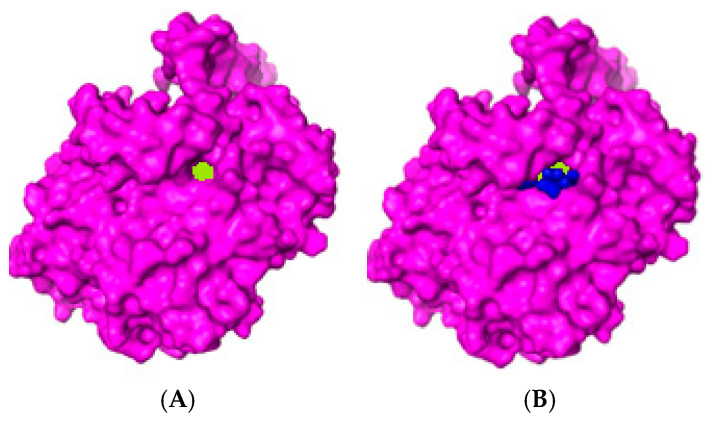
(**A**) Enzyme Cyclooxygenase-2 (COX-2, purple) with its catalytic site (green). (**B**) The blue structure represents the peptide SGHPGAMGPVGPR (P1) interacting with COX-2 active site.

Our research group previously identified a peptide from the bullfrog skin *Lithobates catesbeianus* [14]. The total cyclooxygenase inhibition assay was conducted to validate the predictions from the docking study. Peptide 1 (SGHPGAMGPVGPR) was tested for its ability to inhibit COX activity in the COX inhibition assay. The results showed significant enzymatic inhibition compared to the control (Figure 1). After this analysis, molecular docking was performed to investigate possible interactions between the peptides and cyclooxygenase. Among the peptides analyzed, only the peptide SGHPGAMGPVGPR stood out due to its favorable binding characteristics, and it was named P1. This analysis was conducted using the AutoDockVina© software 1.1.257, with human COX-2 as the receptor. The molecular orientation of Peptide 1 (SGHPGAMGPVGPR) was analyzed based on structural and energetic criteria obtained through molecular docking. COX-2 was selected as the target enzyme because it plays a key role in producing prostaglandins, important mediators of the inflammatory response. The more negative binding free energy values, along with the formation of several hydrogen bonds and hydrophobic interactions with key residues in the catalytic site of COX-2, indicated high affinity and stability of the complex. The positioning of the peptide within the active site supports the hypothesis of its potential inhibitory activity. The molecular docking figure (Figure 2) presents evidence of these interactions, illustrating in detail the peptide-enzyme binding pattern. The interaction energy between the ligand and the COX-2 enzyme is presented in Table 1.

### 2.2. DPPH Radical Scavenging Assay

**Figure 3:**
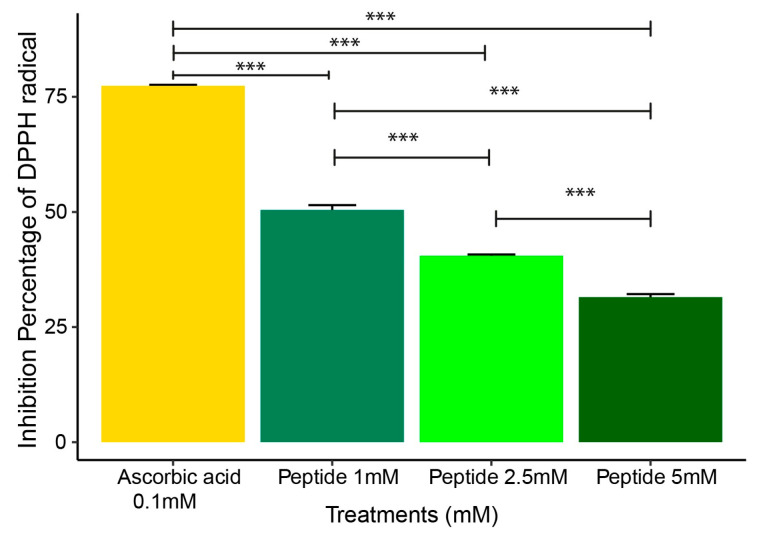
Free radical scavenging activity of peptide 1 at 1 mM, 2.5 mM, and 5 mM was measured using the 2,2-diphenyl-1-picrylhydrazyl (DPPH) assay. Ascorbic acid was used as a reference standard for comparison (0.1 mM). Data are expressed as the mean ± standard error of the mean of three independent assays. One-way ANOVA with Tukey post-hoc test (*** *p* ≤ 0.001).

The free radical scavenging activity of P1 at 1 mM, 2.5 mM, and 5 mM was measured using the 2,2-diphenyl-1-picrylhydrazyl (DPPH) assay. The DPPH assay (2,2-diphenyl-1-picrylhydrazyl) is widely used as a rapid, sensitive, and reproducible method to evaluate the antioxidant capacity of substances, based on the neutralization of free radicals by hydrogen or electron donors [19]. In this study, the assay was performed to determine the ability of the tested compounds to scavenge free radicals, which is a key mechanism in reducing oxidative stress associated with inflammatory processes. The results showed approximately 50% inhibition of the DPPH radical for P1 at 1 mM at 30 min of incubation. The inhibition percentage of DPPH radical was higher for the peptide at 1 mM compared to 2.5 mM and 5 mM (Figure 3).

### 2.3. FRAP Analysis

**Figure 4:**
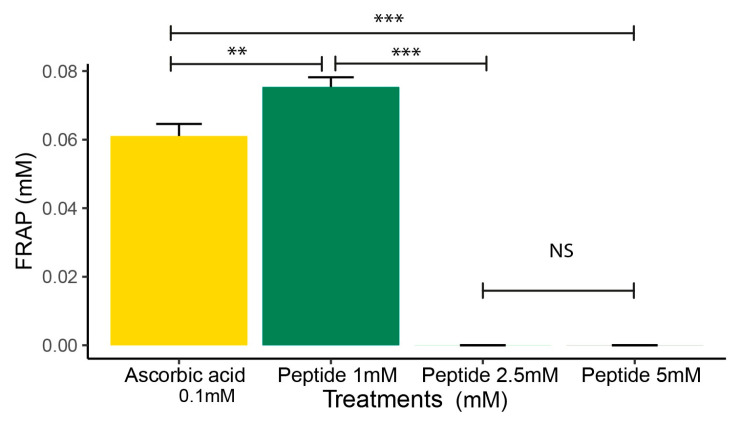
Ferric reducing antioxidant power (FRAP) of peptide 1 at 1 mM, 2.5 mM, and 5 mM. Ascorbic acid was used as the reference standard (0.1 mM). Data are expressed as the mean ± standard error of the mean of three independent assays. A non-parametric Kruskal–Wallis test with Bonferroni correction was conducted (NS—Not significant, ** *p* ≤ 0.01, and *** *p* ≤ 0.001).

The ferric reducing antioxidant power (FRAP) of P1 was measured at 1 mM, 2.5 mM, and 5 mM. The FRAP assay is widely used to evaluate the antioxidant capacity of bioactive compounds, based on the reduction of ferric ion (Fe^3^⁺) to ferrous ion (Fe^2^⁺), detected spectrophotometrically. This method was chosen due to its simplicity and sensitivity, providing an estimate of the reducing capacity and antioxidant potential of the tested peptide [23]. At 1 mM, P1 showed greater antioxidant capacity compared to 2.5 mM and 5 mM. At 2.5 mM and 5 mM, P1 showed negative values (Figure 4).

### 2.4. In Vitro Analysis

#### 2.4.1. Effect of the Peptide on Macrophages Cell Viability

**Figure 5:**
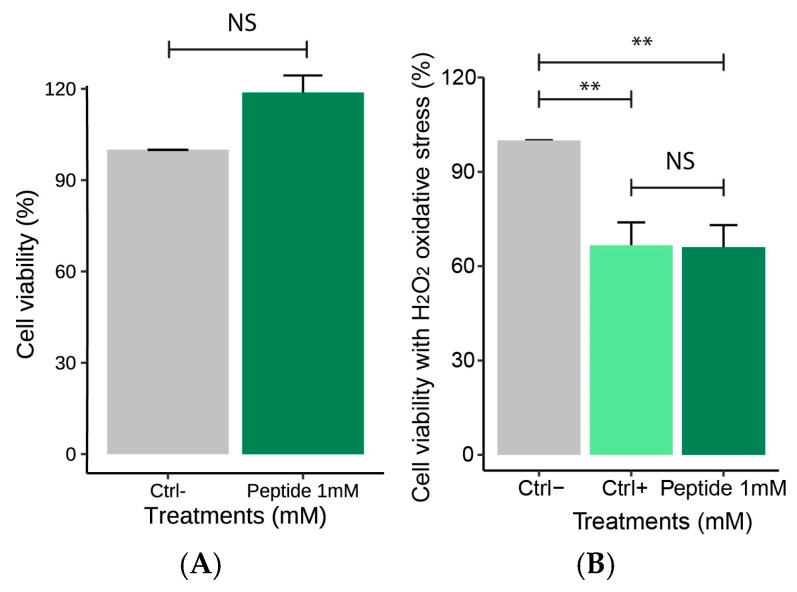
Effect of peptide 1 on RAW264.7 macrophages viability. Macrophages were treated separately with RPMI culture medium and peptide 1. (**A**) Cell viability of Peptide 1 (Cells + RPMI + Peptide1), Ctrl−: Negative control (Cells + RPMI) (*t*-Test, *p* > 0.05). (**B**) Cell viability after hydrogen peroxide-induced oxidative stress, Ctrl−: Negative control (Cells + RPMI), Ctrl+: Positive control (Cell + RPMI + H_2_O_2_) and of the group treated with peptide 1 followed by hydrogen peroxide-induced oxidative stress (Cells + RPMI + Peptide 1 + H_2_O_2_ solution). Data are expressed as the mean ± standard error of the mean of three independent assays. One-way ANOVA with Tukey post-hoc test (NS—Not significant, ** *p* ≤ 0.01).

The cell viability analysis was used to assess the ability of peptide 1 to influence cellular metabolism, allowing the identification of cytotoxic effects [24]. At 1 mM (final concentration of the peptide in the cells), P1 was tested and considered adequate after maintaining macrophages viability above 80%. No significant difference was observed between the negative control and P1 (Figure 5A). After exposing these cells to 1.25 mM hydrogen peroxide, statistical differences were observed comparing P1-treated cells and negative control (Figure 5B).

#### 2.4.2. Effect of the Peptide on Nitric Oxide Production Following H_2_O_2_-Induced Stress

**Figure 6:**
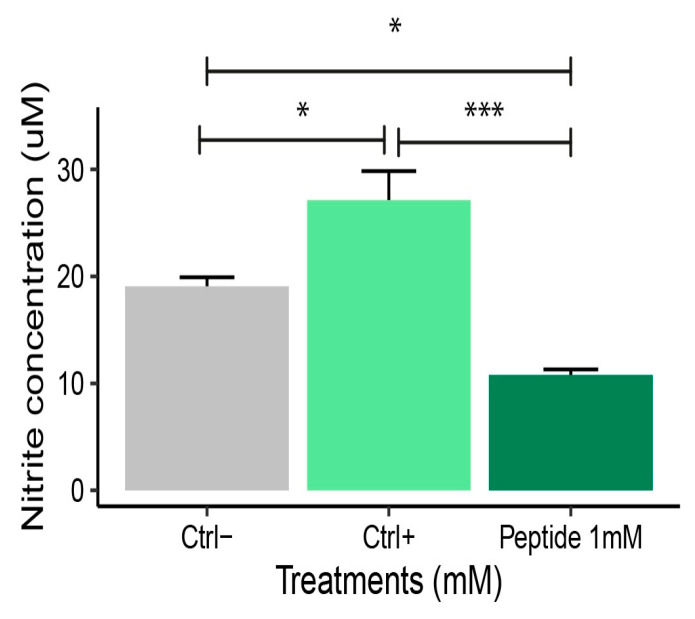
Nitrite levels after RAW 264.7 macrophages treatment with hydrogen peroxide (H_2_O_2_). Macrophages were cultured with RPMI culture medium and treated with peptide 1 after exposure to 1.25 mM H_2_O_2_ for 3 h. Ctrl−: Negative control (Cells + RPMI); Ctrl+: Positive control (Cell + RPMI + H_2_O_2_); Peptide 1: (Cell + RPMI + Peptide 1 + H_2_O_2_ solution). Data are expressed as the mean ± standard error of the mean of three independent assays. One-way ANOVA with Tukey post-hoc test (* *p* ≤ 0.05 and *** *p* ≤ 0.001).

The production of nitric oxide (NO) is directly related to the inflammatory response and oxidative stress [25]. Its quantification allows the evaluation of how these conditions are modulated by bioactive compounds. Therefore, it was included to investigate the anti-inflammatory potential of peptide 1. At 1 mM, P1 significantly reduced NO levels compared to the positive control, demonstrating its ability to mitigate hydrogen peroxide-induced oxidative stress (*p* < 0.05) (Figure 6).

#### 2.4.3. Antioxidant Enzyme Activity (SOD, CAT, GST)

The evaluation of antioxidant enzyme activity, such as superoxide dismutase (SOD), catalase (CAT), and glutathione S-transferase (GST), is essential for understanding the cellular defense mechanisms against oxidative stress. These enzymes act by neutralizing reactive oxygen species, thereby protecting cells from oxidative damage. Analyzing their activity helps determine whether bioactive compounds, such as peptide 1, have relevant antioxidant capacity. Previous studies highlight the importance of these enzymes as markers of oxidative stress [26,27].

**Figure 7:**
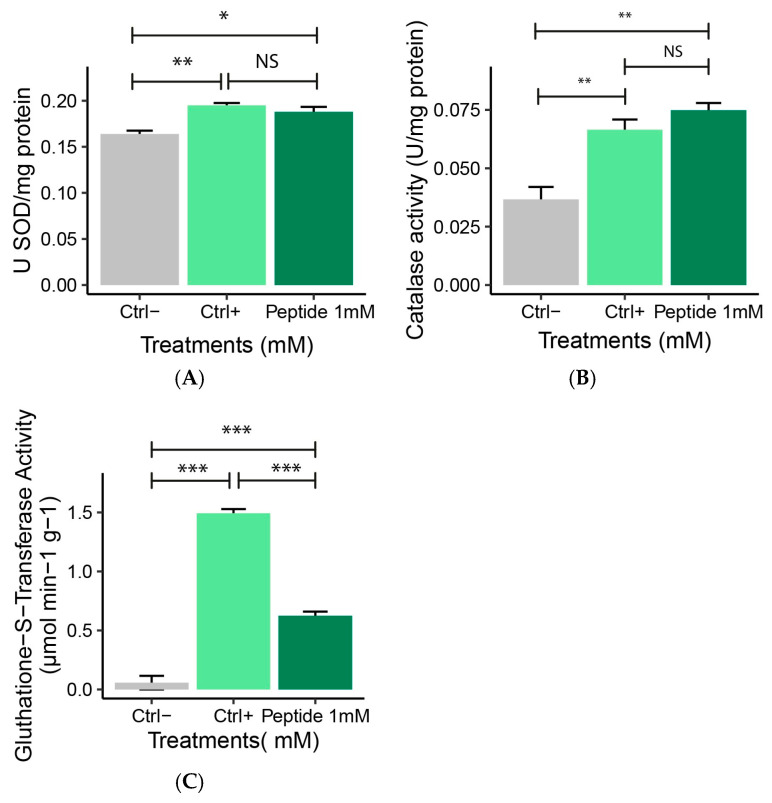
**(A**) Superoxide dismutase, (**B**) catalase, and (**C**) glutathione-s-transferase activity in RAW 264.7 macrophages stressed with hydrogen peroxide (H_2_O_2_) and treated with 1 mM peptide 1. Ctrl−: Negative control: (Cell + RPMI); Ctrl+: Positive control: (Cell + RPMI + H_2_O_2_); Peptide 1: (Cell + RPMI + Peptide 1 + H_2_O_2_ solution). Data are expressed as the mean ± standard error of the mean of three independent assays. One-way ANOVA with Tukey post-hoc test (NS—Not significant, * *p* ≤ 0.05, ** *p* ≤ 0.01, and *** *p* ≤ 0.001).

Superoxide dismutase activity was similar in macrophages treated with 1 mM P1 and positive control (*p* > 0.05) (Figure 7A). There was a difference compared to the negative control, as P1 increased SOD activity (*p* < 0.05). At 1 mM, P1 treatment increased CAT activity in RAW 264.7 macrophages compared to negative controls (*p* < 0.05).

Catalase activity after P1 exposure showed no significant difference compared to positive controls (*p* > 0.05) (Figure 7B). Glutathione activity increased in H_2_O_2_ treated macrophages, as seen in the positive control and P1 groups. At 1 mM, P1 treatment reduced GST activity, showing that the stress produced by H_2_O_2_ was reduced compared to positive control (Figure 7C). The negative control exhibited the lowest GST activity compared to positive control and P1-treated cells.

#### 2.4.4. Multivariate Analysis

**Figure 8:**
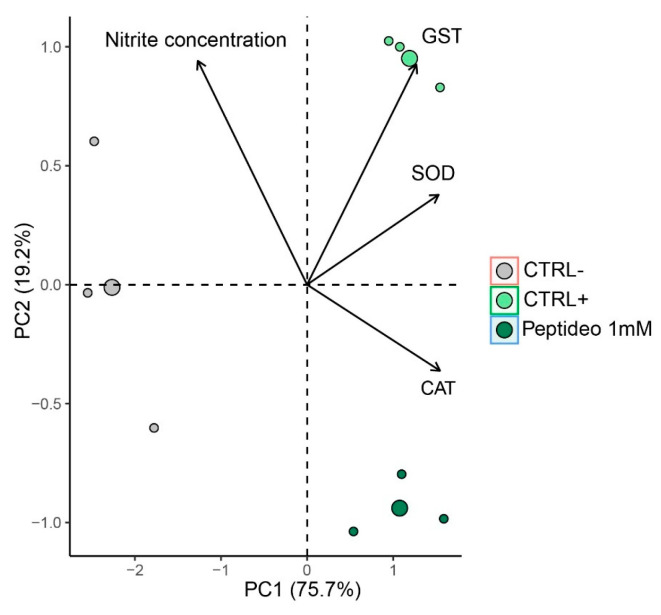
Multivariate analysis based on principal component assay including four variables as follows: catalase activity (CAT), superoxide dismutase activity (SOD), Glutathione-S-Transferase activity (GST), and Nitric Oxide levels (NO). PC1: First principal component; PC2: second principal component. Ctrl−: Negative control: (Cells + RPMI culture medium); Ctrl+: Positive control: (Cell + RPMI culture medium + H_2_O_2_); Peptide 1: (Cell + RPMI culture medium + Peptide 1 + H_2_O_2_ solution).

According to the multivariate analysis, based on oxidative stress data, 94.9% of the total variation was explained using two principal components. The first principal component (PC1) accounted for 75.7.9% of the data variability, while the second principal component (PC2) explained 19.2% (Figure 8). This analysis revealed a clear separation of the samples into three distinct groups, indicating differentiated profiles (Figure 8). Analysis of the variable weights in the principal components identified those that most contributed to the discrimination of antioxidant potential. SOD and GST were the variables most strongly associated with positive control, while CAT showed a stronger relationship with peptides. The negative control is associated with nitric oxide. PCA demonstrated a clear distinction between the negative control, positive control, and peptide groups, with the treatments located in different quadrants (Figure 8), (Permanova: F = 24.549, *p* = 0.00499), (PERMIDISP: F = 1.41, *p* = 0.31), reflecting significant differences in oxidative stress profiles among the analyzed conditions.

### 2.5. Gene Expression Analysis

#### 2.5.1. Expression of Pro-Inflammatory Genes

**Figure 9:**
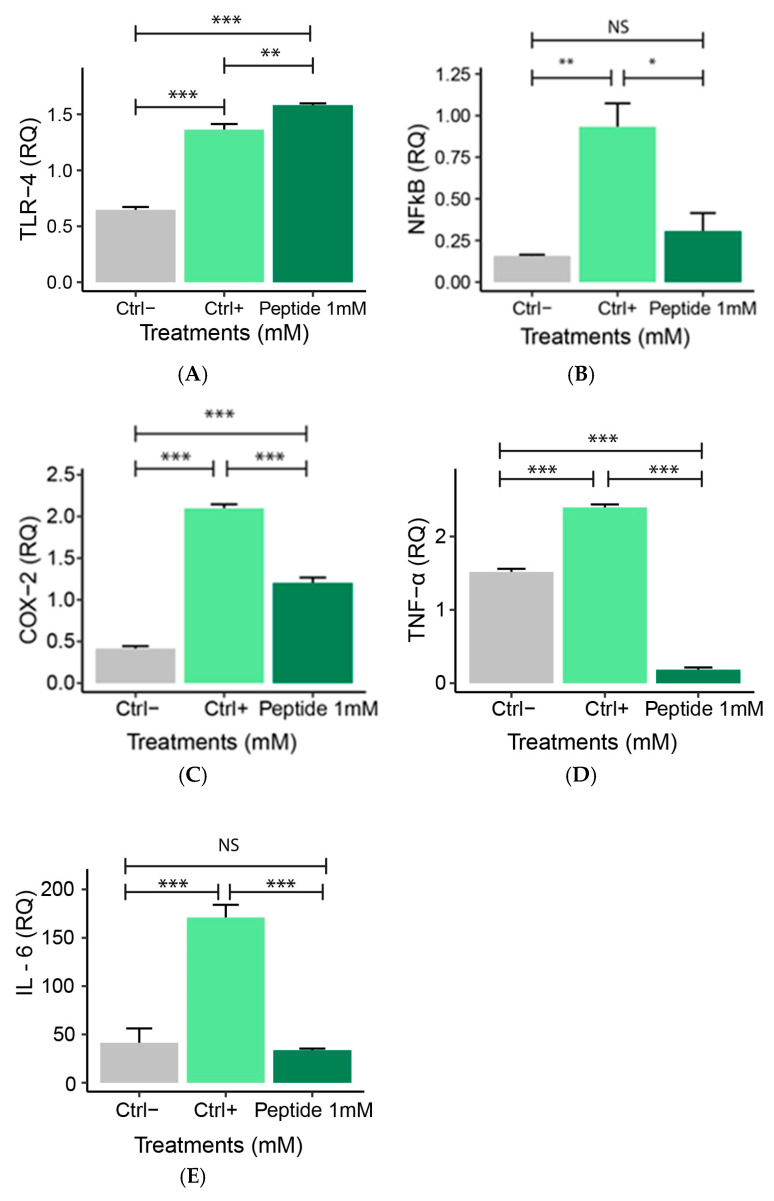
Relative quantification (RQ) of gene expression of pro-inflammatory genes: (**A**) TLR-4, (**B**) NF-κβ, (**C**) COX-2, (**D**) TNFα, and (**E**) IL-6 in RAW 264.7 macrophages treated with peptide 1 for 24 h and stimulated with 10 µg/mL LPS for 4 h. Ctrl−: Negative control: (non-inflamed cells); Ctrl+: Positive control: (cells inflamed with 10 µg/mL LPS), Peptide 1: (Cell + RPMI + Peptide 1 + 10 µg/mL LPS). Data are expressed as the mean ± standard error of the mean of three independent assays. One-way ANOVA with Tukey post-hoc test (NS—Not significant, * *p* ≤ 0.05, ** *p* ≤ 0.01, and *** *p* ≤ 0.001).

Gene expression analysis is essential to understanding the molecular mechanisms underlying the inflammatory response [28]. This analysis was conducted to investigate how peptide 1 may modulate the activation of inflammatory pathways. Relative quantification (RQ) of gene expression allows the measurement of differences in the expression levels of pro- and anti-inflammatory genes [28]. LPS-stimulated macrophages (positive control) presented an increased expression of pro-inflammatory genes (TLR-4, NF-κB, COX-2, TNFα, and IL-6) compared to the negative control. After P1 treatment increased TLR-4 gene expression (Figure 9A) and decreased NF-κB (Figure 9B), COX-2 (Figure 9C), TNFα (Figure 9D), and IL-6 (Figure 9E) gene expression and/or levels were observed compared to positive control.

#### 2.5.2. Expression of Anti-Inflammatory Genes

**Figure 10:**
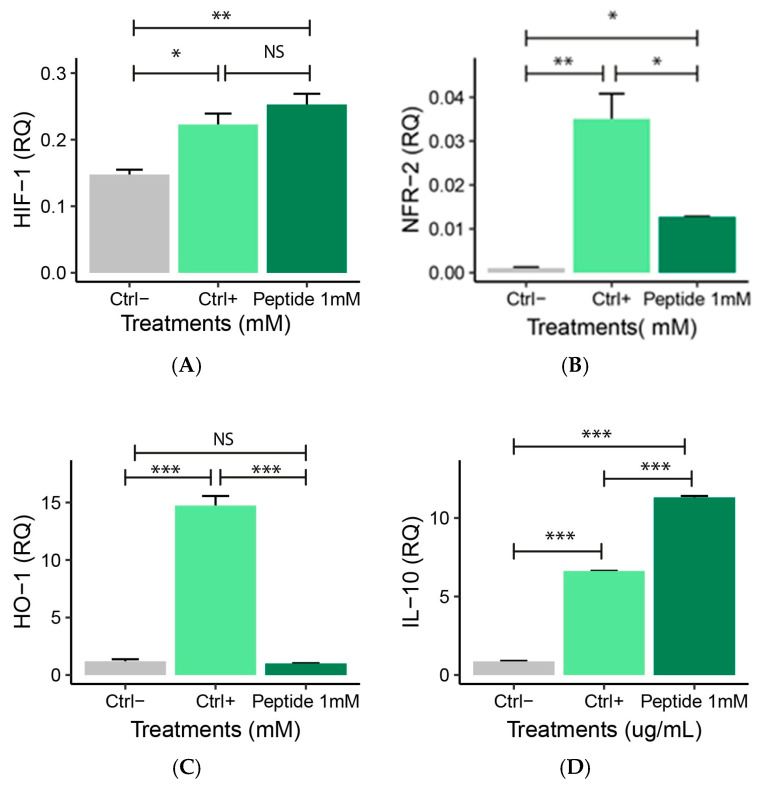
Relative quantification (RQ) of gene expression of anti-inflammatory genes: (**A**) HIF-1, (**B**) Nfr2, (**C**) HO-1, and (**D**) IL-10 in RAW 264.7 macrophage treated with peptide 1 for 24 h and stimulated with 10 µg/mL LPS for 4 h. Ctrl−: Negative control: (non-inflamed cells); Ctrl+: Positive control: (cells inflamed with 10 µg/mL LPS), Peptide 1: (Cell + RPMI + Peptide 1 + 10 µg/mL LPS). Data are expressed as the mean ± standard error of the mean of three independent assays. One-way ANOVA with Tukey post-hoc test (NS—Not significant, * *p* ≤ 0.05, ** *p* ≤ 0.01, and *** *p* ≤ 0.001). For the Nfr2 analysis, a non-parametric Kruskal–Wallis test with Bonferroni correction was conducted.

The analysis of anti-inflammatory gene expression allows the identification of peptide 1’s therapeutic potential in inflammatory diseases. Hypoxia-inducible factor 1-alpha (HIF-1) gene expression (Figure 10A) was similar in P1-treated macrophages and positive control. Furthermore, P1 treatment reduced nuclear factor erythroid 2-related factor 2 (Nfr2) gene expression compared to positive control (Figure 10B). Heme oxygenase-1 (HO-1) expression was reduced after P1 exposure (Figure 10C). Increased IL-10 gene expression was observed in P1-stimulated cells compared to positive control (Figure 10D).

### 2.6. ELISA Analysis

#### IL-6 and TNF-α Levels

**Figure 11:**
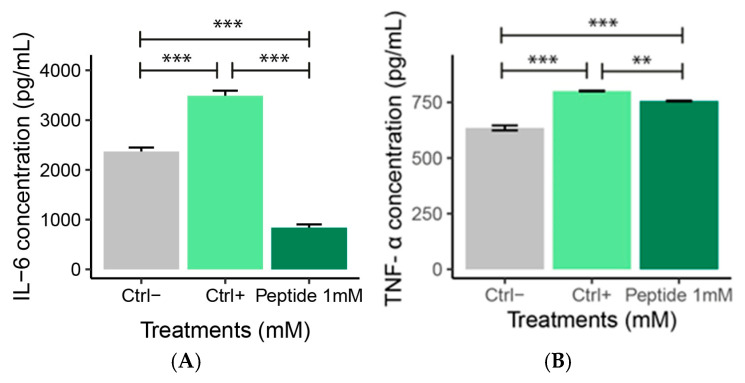
(**A**) IL-6 and (**B**) TNF-α levels after RAW 264.7 macrophages exposure to 1 µg/mL LPS for 4 h. Ctrl−: Negative control (Cells + RPMI); Ctrl+: Positive control (Cells + RPMI + LPS); Peptide 1: (Cells + RPMI + Peptide 1 + LPS). Data are expressed as the mean ± standard error of the mean of three independent assays. One-way ANOVA with Tukey post-hoc test (** *p* ≤ 0.01, and *** *p* ≤ 0.001).

ELISA analysis is widely used to quantify inflammatory cytokines such as IL-6 and TNF-α due to its high sensitivity and specificity [29]. The measurement of these cytokines is essential to understand the activation and regulation of the inflammatory response, as IL-6 and TNF-α play key roles in modulating inflammation and in the development of inflammatory diseases [30]. At 1 mM, P1 treatment reduced the IL-6 levels in LPS-stimulated macrophages compared to positive and negative controls (Figure 11A). Peptide 1 treatment reduced TNF-α levels compared to positive control, which exhibited higher concentrations of this cytokine compared to the negative control (Figure 11B).

### 2.7. Multivariate Analysis

**Figure 12:**
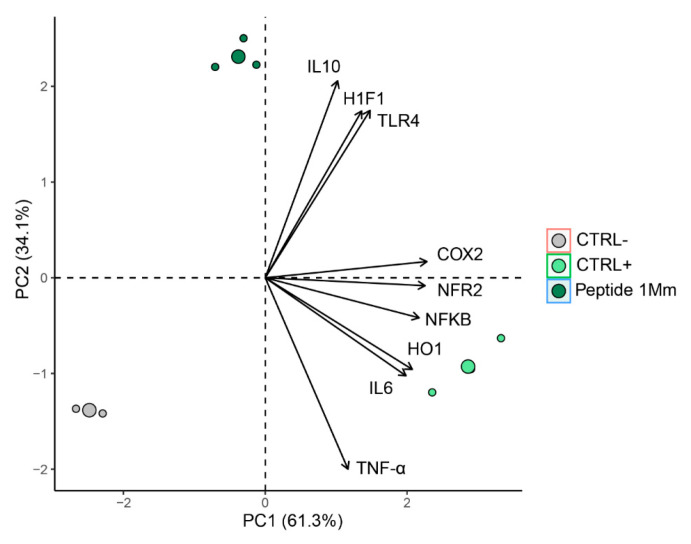
Multivariate analysis based on principal component assay including five inflammatory markers and four anti-inflammatory markers. PC1: First principal component; PC2: second principal component. Ctrl−: Negative control (Cells + RPMI); Ctrl+: Positive control (Cells + RPMI + LPS); Peptide 1: (Cells + RPMI + Peptide 1 + LPS).

**Figure 13:**
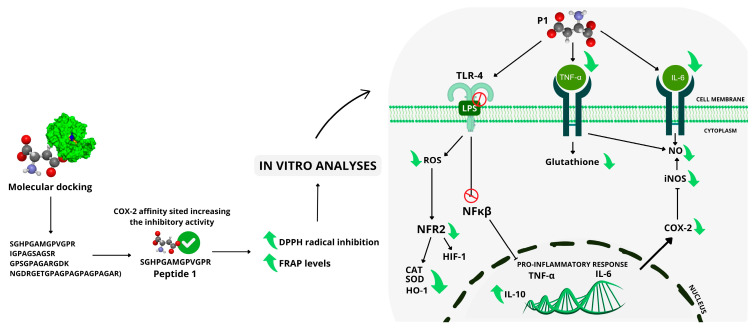
Schematic representation of the effects of Peptide 1 on inflammatory and oxidative stress pathways (OxInflammation). Molecular docking analysis indicated that Peptide 1 has a high affinity for the active site of COX-2, enhancing its inhibitory activity. In vitro assays demonstrated the peptide’s antioxidant potential, reducing free radical formation (DPPH radical inhibition) and increasing antioxidant levels (FRAP levels). Treatment with Peptide 1 resulted in TLR-4 activation, suggesting involvement of the TLR-NF-κB-inflammasome pathway. However, the peptide reduced NF-κB expression and pro-inflammatory cytokines such as TNF-α and IL-6 while inhibiting COX-2 and inducible nitric oxide synthase (iNOS), leading to decreased nitric oxide (NO) production and a reduced inflammatory response. Additionally, the peptide influenced the antioxidant response by modulating the expression of key factors such as Nrf2 and HO-1 (heme oxygenase-1), as well as antioxidant enzymes like SOD (superoxide dismutase) and CAT (catalase), with the latter being analyzed in vitro. The reduction of these markers suggests a regulatory impact on the response to oxidative stress. The reduction in these markers suggests a regulatory effect on oxidative stress response. The interplay between inflammation and oxidative stress (OxInflammation) is highlighted in the figure, indicating that the inhibition of the pro-inflammatory response by Peptide 1 may be linked to the modulation of cellular redox balance. Finally, an increase in the expression of IL-10, an anti-inflammatory cytokine, was observed, reinforcing the immunomodulatory potential of Peptide 1.

Based on gene expression data, 95.4% of the total variation was explained by two principal components. The first principal component (PC1) accounted for 61.3% of the variability, while the second principal component (PC2) explained 34.1% of the total variance (Figure 12). The analysis revealed a clear separation of the samples into three distinct groups (Figure 12), indicating different profiles among the samples. Examination of the variable weights in the principal components identified the variables that most contributed to the discrimination in gene expression. IL-10, HIF-1, and TLR-4 were the variables most strongly correlated with peptide 1 (PC2). The positive control was strongly associated with the variables IL-6, TNF-α, HO-1, and NF-κβ. PCA clearly distinguished between the negative control, positive control, and P1-treated macrophages (Figure 12), with treatments positioned in different quadrants. Data homogeneity within each group was confirmed (homogeneous dispersion) (PERMIDISP: F = 0.7823, *p* = 0.499). Significant differences between treatments were observed Permanova: F = 48.561, R2 = 0.942, *p* = 0.003996. The results obtained are summarized in Figure 13.

## 3. Discussion

Due to their diverse functions and unique properties, animal-derived peptides are crucial in many biological and medical fields. Studies have demonstrated advancements in understanding peptide properties, exhibiting several antibacterial and immunomodulatory activities [21,31]. The structural characteristics of a peptide, such as size, molecular weight, sequence, and amino acid composition, can directly affect its antioxidant and anti-inflammatory activity. Low-molecular-weight peptides (<1.5 kDa) are associated with greater ROS radical scavenging capacity [32,33,34]. When exposed to various environments, amphibian skin requires adequate protection against external damage due to its complex functions and fragility [13]. Skin secretions, especially bioactive peptides from amphibian skin, have been extensively studied and demonstrate biological activities such as antioxidant, reparative, and antibacterial actions [35]. A good example is the Brevinins, a family of antimicrobial peptides found in the skin of frogs belonging to the *Ranidae* family, including brewing-2MP, which is considered a promising therapeutic candidate against bacterial infections due to its potent anti-inflammatory and antimicrobial properties [36]. Consequently, the comprehensive investigation of amphibian-derived peptides can provide valuable information for developing new drugs targeting inflammatory diseases. Based on the promising results obtained by our research group using a natural peptide identified from the bullfrog skin (*Lithobates catesbeianus*) [14], we conducted molecular docking to evaluate the interactions between the enzyme cyclooxygenase-2 (COX-2) and a peptide ligand, predicting its binding mode and affinity. Peptide 1 (SGHPGAMGPVGPR) showed an affinity for the active site of COX-2 and enhanced inhibitory activity (Figure 2). This result is likely to peptide 1 forming more hydrogen bonds, increasing its inhibitory potential. Moreover, the total cyclooxygenase inhibition assay was conducted to validate the predictions from the docking study, in which significant enzymatic inhibition corroborated with the predictions of binding to the active site. Peptide P1 exhibited inhibitory capacity on total COX activity at a concentration of 1 mM, which may be related to specific interactions within its active site (Figure 1).

Based on these findings, we decided to synthesize and purchase the SGHPGAMGPVGPR sequence, which was used in this study to assess its ability to control oxidative stress and the inflammatory process (OxInflammation). Although not clinically detectable, in the OxInflammation process, there is an overlap between oxidative stress and inflammation can induce systemic or local damage over time, exceeding the body’s inherent anti-inflammatory mechanisms and initiating a pathological cycle that exacerbates the progression of various diseases [9]. Oxidative stress is frequently associated with chronic inflammation, where the continuous increase of inflammatory mediators results in the excessive production of reactive oxygen species (ROS) [37,38]. This interaction, when uncontrolled, can cause significant cellular damage. To understand and combat these effects, assessing the antioxidant capacity of substances that can neutralize ROS and interrupt this pathological cycle is essential. Methods such as DPPH and FRAP assays are widely used to measure the antioxidant efficacy of these substances, providing essential data on their ability to eliminate free radicals. In our study, we investigated the antioxidant activity of peptide 1 using DPPH and FRAP assays. The DPPH assay assesses the ability of the peptides to neutralize free radicals generated by superoxide ion (O_2_^·−^), while the FRAP assay measures the reducing power of iron. Our results showed that peptide 1 reduced DPPH levels, achieving about 50% inhibition within 30 min of reaction, demonstrating its antioxidant activity. This suggests the peptide may protect against oxidative damage by capturing free radicals, such as oxygen single and hydrogen peroxide. Similar results were obtained when the peptide OA-GI13 was tested from another amphibian skin. The authors observed that the OA-G13 peptide could eliminate ABTS+ and DPPH free radicals [19]. We can suggest that the decrease in antioxidant activity at higher peptide concentrations may be attributed to potential aggregation effects or conformational changes, which could reduce the availability of active sites for radical scavenging. The antioxidant activity of peptides in the DPPH assay depends on amino acid composition; for example, Cys (cysteine), Met (methionine), and hydrophobic residues enhance activity, while His (histidine) has a negative effect, rather than the sample dose [39]. The antioxidant activity of protein hydrolysates is strongly influenced by structural characteristics such as amino acid composition and peptide molecular weight [40]. Hydrolysates rich in hydrophobic amino acids, such as those obtained from chickpea protein, exhibit high radical scavenging capacity [41]. In the FRAP assay, our results show that peptide 1 presented a greater capacity in reducing ferric ions (Fe^3^⁺) to ferrous ions (Fe^2^⁺) at 1 mM concentration compared to other concentrations. This suggests that the peptide can neutralize reactive oxygen species (ROS) and protect cells against oxidative stress. Therefore, based on the best results obtained for the 1 mM peptide in the DPPH and FRAP assays, all other analyses were conducted with peptide 1 at a concentration of 1 mM. The decrease in antioxidant activity at higher peptide concentrations may be attributed to aggregation effects or conformational changes, which could reduce the availability of active sites for radical scavenging. This protective effect of the peptide may be particularly relevant in reducing the impact of chronic inflammation, helping to prevent further cellular and tissue damage. Our result suggests that the peptide not only acts by directly neutralizing free radicals but can also assist in preserving cellular integrity, particularly in chronic inflammation situations, where ROS levels are elevated.

Our next step was to check the capacity of peptide 1 to influence cellular metabolism through cell viability analyses. This type of analysis is fundamental for studying cellular mechanisms in a controlled manner, allowing for the evaluation of compound efficacy and providing crucial information for developing new therapies, especially in combating oxidative stress and inflammation. The cell viability analysis showed that peptide 1 does not affect cell viability using RAW 264.7 macrophages; on the contrary, it was observed that after exposure to peptide 1, cell viability was maintained above 80%. Similarly, Ref. [42] reported the identification of a short genetic encoding peptide (OA-VI12) in the dermal secretions of *Odorrana andersonii*, which not only preserved cell viability but also stimulated cell metabolism. After observing that peptide 1 was not toxic to the cells by the viability analyses, we tested the capacity to protect the cells against oxidative stress induced by H_2_O_2_ exposure. However, peptide 1 could not protect cells against stress caused by hydrogen peroxide at 1.25 mM. Similarly, the antioxidant peptide AOP-P1, derived from the odorous frog, reduced cell viability after treatment with H_2_O_2_ [43].

Although the peptide showed antioxidant activity in the DPPH and FRAP assays, it was ineffective in protecting cells against H_2_O_2_-induced stress, possibly due to low biological stability or limited cellular penetration. Combining the peptide with bioactive compounds, such as flavonoids and antioxidant vitamins (e.g., vitamin C or E), or metal-chelating agents, could enhance cellular protection, representing a future avenue for exploration. Despite the lack of protective effects of peptide 1 against oxidative stress in this cell viability analysis, our investigation still explored its impact on other cellular processes involved in redox balance. Our results revealed that the production of free radicals was decreased by the inhibition of nitric oxide following treatment with peptide 1. Nitric oxide is a promising candidate due to its significant roles in intracellular signaling, vascular control, and wound healing [44]. In a similar study, neuropeptides isolated from the skin glands of the Stony Creek toad (*Litoria lesser*), called lesueurina, demonstrated inhibition of nitric oxide formation by neuronal nitric oxide synthase (nNOS) [45]. Increased nitric oxide is associated with various microcirculation endothelial cell dysfunction mechanisms, microcirculation changes, and oxidative stress [46]. Therefore, our results indicate that reduced levels of NO help protect against oxidative stress. Following the idea to prove that peptide 1 has an important antioxidant capacity, we evaluated three essential antioxidant enzymes, superoxide dismutase (SOD), catalase (CAT), and glutathione. However, the results showed no significant differences in the activity of these SOD and CAT compared to the control group after oxidative stress promoted by the H_2_O_2_ exposure, suggesting that the 1 mM concentration may not have been sufficient to modulate the antioxidant response effectively or suggesting the activation of the other pathway outside of the activation of the SOD and CAT enzymes. This may be explained by the fact that SOD is a constitutively expressed and highly regulated enzyme, making it less susceptible to changes without prolonged exposure or more severe oxidative stress [47]. Similarly, the catalase activity data, which regulates the breakdown of hydrogen peroxide (H_2_O_2_) by accelerating its conversion into water and oxygen [48], showed no significant difference with treatment. This result suggests that peptide 1 may not act directly on the H_2_O_2_ regulatory pathway or that its antioxidant efficacy may occur through other mechanisms unrelated to catalase activity. It is already known that SOD and CAT can be activated after oxidative stress produced by H_2_O_2_, but it is not the only antioxidant system that can be activated after stressor agent exposure. Following the idea that modulating the antioxidant response may occur through pathways independent of these enzymes, we also analyzed GST activity after peptide 1 exposure. Our results clearly showed that peptide one reduced glutathione levels.

Glutathione acts in the neutralization of ROS such as superoxide radicals [49]. The reduction in glutathione levels suggests that the peptide may prevent oxidative stress caused by H_2_O_2_ exposure, and therefore SOD and CAT were not activated and GST was reduced. If there is no stress in the tissue, there is no need to increase SOD, CAT, and GST activity. One hypothesis is that the peptide’s action leads to a shift in cellular defense systems, causing cells to rely less on GSH, resulting in its reduction. Various factors, including oxidative stress and inflammatory signaling, regulate the expression of γ-glutamylcysteine synthetase (γ-GCS) [50]. Inhibition of TNF-α may reduce γ-GCS expression, leading to lower GSH production [51,52]. This suggests that the peptide’s effectiveness in modulating the antioxidant response may occur through alternative pathways or require prolonged treatment periods to take effect. Therefore, to prove the hypothesis about the influence of the TNF-α activity on GSH production, we decided to do a quantitative ELISA analysis of the influence of peptide 1 on TNF-α. Our results showed a reduction in the TNF-α after peptide 1 exposure, which can help us understand the decrease in the GST levels. Tumor necrosis factor-alpha (TNF-α) is a cytokine with multiple effects on different cell types, recognized as a key regulator of inflammatory and oxidative responses and implicated in the pathogenesis of various inflammatory and autoimmune diseases [53]. It is already known that TNF-α has a crucial role in controlling the oxidative stress and inflammation process, acting in a dual role since stimulation via its second receptor or acting as an alternative pathway to control the oxidative stress. Therefore, the reduction presented by TNF-α activity after peptide 1 exposure also showed the high capacity of peptide 1 to control the inflammation process. To better understand this control, we decided to quantify IL-6, an important cytokine directly involved in the TNF-α production, by activating NF-κB, not the canonical STAT3 pathway.

IL-6 is a pro-inflammatory cytokine that induces the expression of various proteins responsible for acute inflammation and plays a crucial role in cell proliferation and differentiation in humans [54]. IL-6 is produced early in the inflammatory process and causes maturation and activation of neutrophils, maturation of macrophages, and differentiation and maintenance of cytotoxic T cells and NK cells; it also stimulates TNF-α and IL-1β production [55,56,57]. The IL-6 activation/inactivation of monocytes could probably lead to TNF-α production/inhibition through the activation/inactivation of NF-κB [58,59]. Our results showed that after peptide 1 exposure, there was a reduction in IL-6, TNF-α, and NF-κB expression. The IL-6 and TNF-α activity reduction confirmed the molecular analyses using ELISA analyses. Therefore, our results showed that peptide 1 can control the OxInflammation process by reducing the IL-6, TNF-α, and NF-κB pathways. These findings are consistent with previous studies where the synthetic peptide (OA-GP11d), initially identified from the skin secretion of the odoriferous toad, inhibited the release of inflammatory factors associated with a reduction in the TNF-α and IL-6 [60]. Additionally, the NCWPFQGVPLGFQAPP peptide exhibited anti-inflammatory effects in LPS-stimulated RAW264.7 macrophages by inhibiting TNF-α and IL-6 [61].

To understand other possible pathways involved in the regulation of the OxInflammation process after peptide 1 exposure, we quantified beyond IL-6, TNF-α, and NF-κB, as cited above, other key genes related to inflammation and immune response, through the regulation of the oxidative stress. Therefore, we quantified TLR-4, COX-2, HIF-1, Nfr2, HO-1, and IL-10, and some of these genes are involved in the inflammasome pathways. Our results revealed an increase in TLR receptor 4 levels, a significant reduction in NF-κB expression, and a reduction in other pro-inflammatory markers such as COX-2, TNF-α, and IL-6. Additionally, an increase in the expression of the anti-inflammatory marker IL-10 was observed. In this case, our hypothesis is: Although peptide 1 increased the expression of the TLR4 receptor, our findings show that peptide 1 (SGHPGAMGPVGPR) can inhibit TLR4 activation caused by LPS, blocking the classical inflammatory cascade and favoring a regulatory response. Similar studies, such as reference, support this hypothesis [62], which demonstrated that the SPA4 peptide interacts with TLR4 and attenuates the LPS-induced inflammatory response, while [63] reported that the Andersonin-W1 peptide modulates the NF-κB pathway, reduces LPS binding, and promotes a balanced healing environment. Similarly, Ref. [64] revealed in a study that peptides selectively inhibit TLR4 receptor signaling by blocking the interaction between TLR4/MD2 and LPS, thus hindering LPS access to MD2. This action results in reduced IκB degradation, delayed nuclear translocation of NF-κB, and inhibition of MAPK pathway activation, decreasing the secretion of pro-inflammatory cytokines, such as IL-6, and reducing oxidative stress markers, including NO and ROS. These findings suggest that by increasing TLR4 expression and reducing inflammatory signaling, the tested peptide exerts a complex regulatory effect on inflammatory signaling.

Regulatory and antioxidant mechanisms, including NrF2, HO-1, and HIF-1, are activated by oxidative stress. These mechanisms stimulate the expression of antioxidant enzymes that neutralize free radicals and reduce inflammation [19]. Upon activation by stress signals detected by the KEAP1 protein, Nrf2 translocates to the nucleus, where it binds to antioxidant response elements (AREs) in the promoters of target genes, thereby initiating their transcription [19]. Consequently, there is an increase in the expression of antioxidant enzymes, such as HO-1, SOD, and CAT [65]. HO-1 is crucial for defending against oxidative stress and reducing inflammation [65]. Our results showed that there was a reduction in Nrf2/HO-1 after exposure to peptide 1, which may help us understand why it did not stimulate the production of the antioxidant enzymes SOD and CAT. This suggests that the efficacy of the peptide in modulating the antioxidant response may be related to pathways distinct from those dependent on Nrf2/HO-1 or require higher concentrations and prolonged treatment periods to show an effect. Nrf2/HO-1 is a crucial antioxidant pathway for the response to oxidative stress, but its regulation is highly dynamic and can be influenced by various factors, such as the intensity of stress and the inflammatory response. Therefore, the reduction of Nrf2/HO-1 after exposure to peptide 1 suggests a cellular adaptation that prioritizes other antioxidant-protection pathways. Furthermore, regulation of the Nrf2 pathway can influence other signaling pathways, such as HIF-1, which is involved in the cellular response to oxidative stress and hypoxia [66]. Hypoxia-inducible factor-1 (HIF-1) is a transcription factor that regulates oxygen (O_2_) homeostasis and binds to hypoxia response elements (HREs) to activate the transcription of several genes in response to low O_2_ [3]. Regarding the expression of the HIF-1 gene, no significant differences were observed compared to the control after exposure to peptide 1 at a concentration of 1 mM, which may help us understand that the reduction of Nrf2 may have influenced this result. We suggest that multiple signaling pathways may regulate HIF-1, and the peptide may be affecting other pathways that, directly or indirectly, influence HIF-1 expression, resulting in a complex effect that is not directly reflected in the analyzed expression levels. Furthermore, there is a growing body of evidence indicating that Nrf2 signaling contributes to the activation and maintenance of the HIF-1 response, and several studies have shown that the reduction of Nrf2 is sufficient to reduce HIF-1α at the post-translational level [67,68,69]. Therefore, our hypothesis is that our result may be a consequence of the homeostatic adjustment between these pathways, as Nrf2 and HIF-1 share activation and inhibition mechanisms under stress conditions.

These findings have important implications for developing therapies for inflammatory and oxidative stress conditions and highlight the need for further studies to fully understand the mechanisms of action and efficacy of peptide 1.

## 4. Materials and Methods

### 4.1. Peptide Synthesis and Molecular Docking

Our group tested one peptide sequence obtained from bullfrog skin (*Lithobates catesbeianus*) and presented the biological effects on cell viability. In previous studies, this peptide sequence was identified from 71 peptide sequences obtained from fraction F4 derived from bullfrog skin—*Lithobates catesbeianus*—through mass spectrometry [14]. This peptide was used to perform COX inhibition assay and molecular docking analyses using human COX-2 as the receptor. AutoDockVina© 1.1.257 software, following a modified methodology, was also used. Human COX-2 (PDB ID: 5IKR) was used as the receptor, with necessary editing (removal of water molecules, addition of non-polar hydrogens, and protein charge calculation) done using AutoDockTools. The receptor file was converted to PDBQT format. The ligand and the peptide developed in this study were designed using the software Marvin Sketch 17.28.049, with all hydrogen atoms explicitly shown. Molecular docking determined its optimal interaction with COX-2. Therefore, it was synthesized and purchased from Aminotech Indústria e Comércio Ltd.a. Sorocaba, SP, Brazil, CNPJ: 12.762.123/0001-49, nº 000756, Molar Mass 1219.28, purity above 90%, and on a 10 mg scale. Mass spectrometry was performed using the Shimadzu LC/MS 20/20 system (Kyoto, Japan) to determine the molecular mass of the peptide (see Supplementary Material). The purity of SGHPGAMGPVGPR was detected by high-performance liquid chromatography (HPLC) (HPLC Shimadzu 6AD, Kyoto, Japan), following the protocol described in a previous study (see Supplementary Material) [70]. After synthesis, the peptide was resuspended in ultrapure water, free of RNase and DNase, ensuring the purity and integrity of the samples for subsequent assays. The peptide SGHPGAMGPVGPR concentrations (1 mM, 2.5 mM, and 5 mM) were prepared from a 50 mM stock solution for DPPH and FRAP assay testing.

### 4.2. DPPH Radical Scavenging Assay

After synthesis, the peptide was resuspended in ultrapure water, free of RNase and DNase, to ensure the purity and integrity of the samples. The peptide solution was then added to the DPPH solutions for evaluation of antioxidant activity. The antioxidant activity was determined by the reduction of the DPPH radical, which converts to diphenyl picrylhydrazine, a yellow compound, in a reaction that stabilizes after 30 min [71,72]. The peptide concentrations (1 mM, 2.5 mM, and 5.0 mM) were prepared in methanol PA, 99% (Merck, Darmstadt, Germany). Ascorbic acid was used as a reference standard (0.1 mM) due to its well-established ability to donate electrons and neutralize free radicals, ensuring direct comparison and validation of the antioxidant activity of the tested compound [71]. An aliquot of 50 µL for each peptide concentration and 250 µL of DPPH solution was added to each well. The samples were analyzed at 0 and 30 min of incubation in the dark in a microplate reader at 517 nm. The ability of the peptide to reduce the radical was calculated as follows.

% Inhibition = (ADPPH − APEPTIDE)/ADPPH) × 100

where ADPPH is the absorbance of the DPPH solution, and APEPTIDE is the absorbance of the sample.

### 4.3. FRAP Analysis

The total antioxidant capacity was estimated using the ferric-reducing ability of plasma (FRAP) method as described by [23], employing TPTZ (2,4,6-Tris(2-pyridyl)-s-triazine) as the substrate. This method reduces a ferric 2,4,6-tripyridyl-s-triazine complex (Fe^3^⁺ + -TPTZ) to its ferrous form (Fe^2^⁺ + -TPTZ). After synthesis, the peptide was resuspended in ultrapure water, free of RNase and DNase, to ensure the purity and integrity of the samples. The peptide solution was then added to the DPPH solutions to evaluate of antioxidant activity. Samples (10 μL) of the peptide SGHPGAMGPVGPR at concentrations of 1 mM, 2.5 mM, and 5 mM were added to the FRAP solution (190 μL), which consisted of 25 mL of acetate buffer (300 mmol/L, pH 3.6), 2.5 mL of TPTZ reagent (10 mmol/L), and 2.5 mL of FeCl_3_·6H_2_O solution (20 mmol/L). The increase in absorbance at 593 nm was measured to determine the Fe^3^⁺ + -TPTZ complex reduction by antioxidants in the samples. The reducing capacity was quantified using a standard curve prepared from serial dilutions of FeSO_4_·7H_2_O starting at 1 mmol/L. The results were expressed as FRAP values. A concentration of 0.1 mM of ascorbic acid was used as the reference standard for comparison [73]. The concentrations for the other tests were also determined based on the antioxidant results from DPPH and FRAP.

### 4.4. In Vitro Analyses

#### 4.4.1. Cell Viability

The cell viability of RAW 264.7 (ATCC^®^ No. TIB-71™) macrophages was assessed using the 3-[4,5-dimethylthiazol-2-yl]-2,5-diphenyl tetrazolium bromide (MTT) assay as previously described [56,74]. The murine macrophage cell line RAW 264.7 (ATCC TIB-71) was purchased from the American Type Culture Collection (ATCC; Manassas, VA, USA). The MTT assay measures cellular metabolic activity to indicate cell viability, and cytotoxicity. This colorimetric assay is based on the reduction of the yellow tetrazolium salt (3-[4,5-dimethylthiazol-2-yl]-2,5-diphenyl tetrazolium bromide or MTT) to purple formazan crystals by metabolically active cells. The cells were cultured in RPMI supplemented with 10% fetal bovine serum and 0.1 g/L penicillin/streptomycin in a humidified incubator with 5% CO_2_ at 37 °C for 24 h. RAW 264.7 macrophages were cultured in 96-well plates at a density of 2 × 10^4^ cells/well in 200 µL of medium. After 24 h, 1 mM peptide (final concentration of the peptide in the cells) was added, and the cells were incubated at 37 °C with 5% CO_2_ for an additional 24 h. The control (100% growth) consisted of cells cultured only in a medium (RPMI supplemented with 10% fetal bovine serum and antibiotics), without the addition of peptide or any extra solvent. Subsequently, the 50 µL MTT solution (0.5 mg/mL) was added to each well, and the cells were incubated for 1 h at 37 °C. The formazan formed during the incubation was dissolved in 100 µL DMSO, and the absorbance was measured at 570 nm. The result was expressed as a percentage of absorbance relative to the control group.

#### 4.4.2. Cell Viability After Induction of Oxidative Stress with Hydrogen Peroxide

RAW 264.7 macrophages were subjected to oxidative stress induced by hydrogen peroxide. After 24 h, a cell viability test was performed to evaluate the concentration (in molarity) at which hydrogen peroxide had deleterious effects. A concentration curve was constructed to determine the dose that resulted in an 80–90% reduction in cell viability. With the harmful concentration of H_2_O_2_ defined, other RAW 264.7 macrophages were incubated (37 °C and 5% CO_2_) for 24 h and treated with the peptide at a concentration of 1 mM. After incubation, the peptide-treated cells were exposed to the chosen toxic concentration, fixed at 1.25 mM for 3 h. This concentration was selected based on preliminary toxicity assays, in which different concentrations of H_2_O_2_ were tested to determine the point at which cell viability was significantly reduced without causing immediate cell lysis. This approach allowed the selection of an appropriate dose to evaluate the protective capacity of the peptide against oxidative stress. The protective effect of the peptide after exposure to H_2_O_2_ was evaluated using the MTT assay (3-(4,5-dimethylthiazol-2-yl)-2,5-diphenyltetrazolium bromide).

#### 4.4.3. Superoxide Dismutase (SOD) Analysis

RAW 264.7 macrophages were cultured in 96-well plates at a density of 4 × 10^5^ cells/well, with 200 μL of medium per well. After 24 h of incubation at 37 °C with 5% CO_2_, the peptide was added at a concentration of 1 mM, and the cells were incubated for an additional 24 h. Oxidative stress was then induced by treatment with 1.25 mM hydrogen peroxide (H_2_O_2_) for 3 h. After this period, the medium was discarded, and the cells were lysed using PBS containing 1% Triton X-100. The cell extracts were stored at −80 °C for subsequent antioxidant activity analysis. For the analysis of superoxide dismutase (SOD) activity, the frozen extracts were thawed and used directly in the assays. This prior freezing aimed to preserve cellular components until biochemical analysis. For the SOD enzymatic assay, MTT (1.25 mM) and pyrogallol (0.1 mM) solutions were prepared in 0.2 M phosphate buffer (pH 8.0). The assay was carried out in a new 96-well plate as follows: Blank wells: received 144 μL of phosphate buffer and 6 μL of MTT solution (no sample and no pyrogallol; used to eliminate optical interference from reagents and buffer); Control wells: received 129 μL of phosphate buffer and pyrogallol, but no sample (no cellular extract); Test wells: received 30 μL of the cell extract (sample) and 99 μL of phosphate buffer (used to maintain the pH of the reaction). All wells received 6 μL of the MTT solution. Then, 15 μL of the pyrogallol solution was added to all wells except the blanks. The plates were incubated for 15 min at 40 °C. The reaction was stopped by adding 150 μL of DMSO to each well. Absorbance was measured using a microplate spectrophotometer at 540 nm. SOD activity was determined based on the reduction in formazan formation, indicating higher antioxidant capacity in the treated samples. Enzymatic activity values were calculated as the ratio between the sample absorbance and the control absorbance, both previously corrected by the average absorbance of the blank wells (which contained neither sample nor pyrogallol). The formula used was: (Sample absorbance − blank)/(Control absorbance − blank). This ratio reflects the relative activity of the SOD enzyme based on the inhibition of pyrogallol oxidation [75].

#### 4.4.4. Catalase (CAT) Activity

RAW 264.7 macrophages were cultured at a density of 1 × 10^6^ cells/well in six-well plates and incubated for 24 h. The difference in the number of cells used for the catalase (CAT) and superoxide dismutase (SOD) activity assays is due to the specific requirements of the protocols used for each enzyme [75,76,77]. For CAT activity, the protocol required a higher number of cells to ensure an adequate enzyme quantity for accurate detection, while the SOD protocol allowed for analysis with a smaller number of cells. After 24 h, the cells were treated with 1 mM peptide for 24 h and then exposed to a 1.25 mM H_2_O_2_ solution for 4 h. The treatments were divided as follows: Non-stressed control (CTRL−): cells were treated only with RPMI culture medium for 24 h; Stressed control (CTRL+): cells were treated with RPMI culture medium for 24 h and then exposed to a predetermined concentration of H_2_O_2_ for 4 h; Non-stressed treatment: cells were treated with the peptide at a concentration of 1 mM for 24 h without H_2_O_2_ exposure; Stressed treatment (Peptide 1): cells were treated with 1 mM peptide for 24 h, followed by the exposure to a predetermined concentration of H_2_O_2_ for 4 h. After incubation, the culture medium was removed, and the cells were washed with PBS before being resuspended in the culture medium. The samples were centrifuged at 2000 rpm for 10 min at 4 °C. The pellets were resuspended in 1 mL lysis buffer (50 mM potassium phosphate buffer, pH 7.0; 0.25% Triton X-100; 1 mM EDTA) and homogenized using a vortex. Catalase activity was assessed by measuring the absorbance at 240 nm in a UV 96-well plate, where a reduction in absorbance indicates the consumption of H_2_O_2_ by the catalase enzyme [76,77]. The protein content of the samples was measured according to Bradford 1976 [78].

#### 4.4.5. Glutathione (GST) Analysis

RAW 264.7 macrophages were cultured in 96-well plates at a density of 4 × 10^5^ cells/well, with 200 μL of medium per well. After 24 h of incubation at 37 °C with 5% CO_2_, the peptide was added at a concentration of 1 mM, and the cells were incubated for an additional 24 h. Oxidative stress was then induced by treatment with 1.25 mM hydrogen peroxide (H_2_O_2_) for 3 h. After this period, the medium was discarded, and the cells were lysed using PBS containing 1% Triton X-100. The cell extracts were stored at −80 °C for subsequent antioxidant activity analysis. For the analysis of Glutathione (GST) analysis, the frozen extracts were thawed and used directly in the assays. The analysis was performed by adding 970 µL de phosphate buffer pH 7, 10 µL de 1-chloro-2,4-dinitrobenzene (CDNB), 10 µL of cell lysate, and 10 µL de reduced glutathione (GSH) in a quartz cuvette, and the absorbance was measured at 0, 30, 60, and 90 s [79,80]. For comparison, a blank was prepared similarly, replacing the sample with a buffer. The values plotted along the Y-axis in the graph represent glutathione concentrations, which were calculated based on the average of values obtained from triplicate measurements. The Y-axis scale, ranging from 0.0 to 1.5, was used to represent the variation in glutathione activity. The result was expressed in umol.min^−1^. g^−1^, calculated by the difference between the final and initial time, according to the following formula: (Abs sample (T90 − T0) − Abs control (T90 − T0)) × 104.17. The formula calculates the difference in GST activity between the treated sample and the control, correcting for absorbance variation over the 90-s reading period. This is done to compare the enzymatic activity under the tested conditions to the baseline (control), and the conversion factor adjusts the unit to the amount of product formed per minute per gram of sample. The difference in absorbance readings between the sample and control at the 90-s (T90) and initial (T0) times provides a measure of enzyme activity, with the conversion to the appropriate unit. The value of 104.17 is a conversion factor for the enzyme activity unit used in the study. The absorbance was measured at 340 nm using a plate spectrophotometer, as the CDNB-GSH conjugate exhibits an absorption peak at this wavelength. This allows for the quantification of enzymatic activity [79,80].

#### 4.4.6. Nitric Oxide (NO) Quantification

RAW 264.7 macrophages were cultured in 96-well plates at a density of 4 × 10^5^ cells/well, with 200 μL of medium per well. After 24 h of incubation at 37 °C with 5% CO_2_, the peptide was added at a concentration of 1 mM, and the cells were incubated for an additional 24 h. Oxidative stress was then induced by treatment with 1.25 mM hydrogen peroxide (H_2_O_2_) for 3 h. After this period, the medium was discarded, and the cells were lysed using PBS containing 1% Triton X-100. The cell extracts were stored at −80 °C for subsequent antioxidant activity analysis. For the analysis of Nitric Oxide (NO) Quantification, the frozen extracts were thawed and used directly in the assays [81]. A standard curve of sodium nitrite solution (0–0.25 mM) was prepared, and the analysis was performed in triplicate to ensure the accuracy of the result. Thereafter, 50 µL of cell lysate (intracellular content released after the lysis of treated cells) or different concentrations of sodium nitrite solution (standard solutions with known concentrations of sodium nitrite: (0.0039, 0.0078, 0.015, 0.03, 0.06, 0.05, 0.125, 0.25 mM) used to generate the standard curve) were added to the 96-well plate, and 100 µL of sulfanilamide and naphthyl ethylenediamine solution (1:1) were added. The solution was incubated in the dark for 10 min to allow for the complete formation of the azo complex (colored compound formed during the Griess reaction). After incubation, the intensity of the color of the azo compound was measured using a spectrophotometer at 570 nm. The values related to nitric oxide (NO) production were obtained indirectly by quantifying nitrite (NO_2_^−^) levels in the samples using the Griess method. For quantification, a standard curve of sodium nitrite (0 to 0.25 mM) was used, and the sample values were interpolated based on this curve. Thus, the enzymatic activity related to NO production was expressed in terms of nitrite concentration (mM), allowing for an indirect estimate of nitric oxide release by the treated cells.

### 4.5. Gene Expression Analysis

The gene expression of pro- and anti-inflammatory cytokines was determined by quantitative reverse transcription PCR (qRT-PCR) analysis [28,82]. Macrophages were cultured at a density of 2.5 × 10^5^ cells/well in six-well plates and treated with RPMI medium supplemented with 10% fetal bovine serum (FBS) and 1 mM peptide. The cells were incubated at 37 °C in a 5% CO_2_ atmosphere for 24 h. After this period, the macrophages were exposed to 10 µg/mL of LPS (lipopolysaccharide) for 4 h to induce inflammation [28]. Subsequently, the cells were harvested, and total RNA was extracted using TRI Reagent^®^ (Sigma-Aldrich, St. Louis, MO, USA). LPS is used to induce acute inflammation by activating TLR4 receptors in macrophages [83]. The RAW 264.7 cell line was chosen for its ability to exhibit functional plasticity, allowing the study of the inflammatory and oxidative response to the peptide. Therefore, LPS and macrophages allow for understanding the mechanisms activated after exposure to peptides to control acute inflammation and, consequently, oxidative stress following the respiratory burst. This enables the evaluation of the modulation of pro- and anti-inflammatory cytokines by the peptide and investigation of its potential immunomodulatory effects. The RNA samples were stored in 1.5 mL microtubes at −80 °C until processing. RNA was isolated according to the manufacturer’s protocol, and its concentration and quality were assessed using a Multiskan SkyHigh spectrophotometer (Thermo Fisher Scientific, Waltham, MA, USA) on a μDrop Duo plate. The extracted RNA (1000 ng) was then reverse transcribed into cDNA using the High-Capacity cDNA Reverse Transcription Kit (Thermo Fisher Scientific). RT-qPCR quantification quantified with PowerTrack™ SYBR™ Green Master Mix (Thermo Fisher Scientific) on a QuantStudio™ 3 system (Thermo Fisher Scientific). Beta-actin was used as the reference gene, and the relative standard curve method was employed for quantitative data analysis. Controls included the negative control (Ctrl−) with mRNA from non-inflamed macrophages and the positive control (Ctrl+) with mRNA from LPS-inflamed macrophages. The primers used are listed in Table 2.

Table 2: Mouse primer sequences: COX-2: Cyclooxygenase; NF-κβ: Nuclear factor kappa B; IL-6: Interleukin 6; TNF-α: Tumor necrosis factor-α; TLR: Toll-like receptors; HIF-1: Hypoxia-inducible factor 1-alpha; IL-10: Interleukin-10.

### 4.6. ELISA Analysis

#### Murine IL-6 and TNF-α Quantification

To quantify IL-6, the Murine IL-6 (PeproTech, Waltham, MA, USA, Cat. No. 900-M50) and TNF-α, an ELISA kit (Invitrogen, Waltham, MA, USA, Cat. No. 88-73224), were used according to the manufacturer’s instructions. The microtiter plate was coated with 100 µL of capture antibody diluted in PBS (2 µg/mL) and incubated overnight at 4 °C. After four washes with Wash Buffer (PBS + 0.05% Tween-20) and blocking with 300 µL of block buffer containing 1% BSA for 1 h at room temperature, 100 µL standard dilutions and 100 µL of cell culture samples (5 × 10^5^ cells) were added per well and incubated again overnight at 4 °C. The detection antibody, diluted in PBS + 1% BSA, was added to each well and incubated for 2 h at room temperature, followed by three washes with PBS-T. Streptavidin-HRP conjugate was added, incubated for 30 min, and the plate was rewashed. The reaction was developed with 100 µL of ABTS substrate, incubated until colour development (5–30 min), and stopped with 100 µL of Stop solution (1N sulfuric acid). Absorbance was measured at 405 nm. The concentrations of TNF-α in the samples were determined based on the standard curve of TNF-α, and IL-6 concentrations were determined based on the standard curve of IL-6 (0–4000 pg/mL). The cells were stressed with LPS for 4 h to perform this analysis.

### 4.7. Statistical Analysis

The experimental design used was completely randomized. Data were subjected to normality analysis using the Shapiro–Wilk test and variance homogeneity analysis using Bartlett’s test. Data were then analyzed using analysis of variance (ANOVA), followed by mean comparisons with the Tukey test. *p*-values > 0.05 were considered non-significant. Cell viability was compared between the control (RPMI/10%) and the treatment with 1 mM peptide using the *t*-test. The data were analyzed using Principal Component Analysis (PCA) and Permutational Multivariate Analysis of Variance (PERMANOVA), with 1000 permutations and Euclidean distance. All statistical analyses were performed using R software version 4.3.3 [84]. For the Nfr2 analysis, a non-parametric Kruskal–Wallis test with Bonferroni correction was conducted.

## 5. Conclusions

The synthetic peptide SGHPGAMGPVGPR demonstrated promising properties in the modulation of oxidative stress and inflammation. This exploratory study demonstrated, for the first time, the anti-inflammatory and antioxidant effects (OxInflammatory process) of peptide 1 (SGHPGAMGPVGPR). However, to determine a mechanistic pathway, more in-depth experiments are necessary. The data obtained provided a mechanistic basis to explain how this peptide can control the OxInflammatory process and may be used in the future as a tool for the development of new therapies for the treatment of inflammatory diseases. These promising results open possibilities for future investigations to understand the influence of molecular pathways on tissue function by testing this peptide in human tissue (ex vivo). Therefore, our results provide new insights into the relationship between oxidative stress and the inflammatory process.

## Figures and Tables

**Table 1:** The interaction energy between the ligand (P1) and the cyclooxygenase 2 (COX-2) enzyme.

Peptide	Origin Protein	COX-2 Binding Energy	COX-2 Inhibition Active Site
SGHPGAMGPVGPR	Collagen α-2 (I) *	−9.5 (kcal/mol)	3

* Collagen alpha-2 (I) chain.

**Table 2:** Primers Sequence.

Gene	Forward	Reverse
NF-κβ	5′-GCT GCC AAA GAA GGA CAC GAC A-3′	5′-GGC AGG CTA TTG CTC ATC ACA G-3′
COX-2	5′-TGC ACT ATG GTT ACA AAA GCT GG-3′	5′-TCA GGA AGC TCC TTA TTT CCC TT-3′
IL-6	5′-TCC TTC CTA CCC CAA TTT CC-3′	5′-GCC ACT CCT TCT GTG ACT CC-3′
TNF-α	5′-TAT GGC TCA GGG TCC AAC TC-3′	5′-CCC ATT TGA GTC CTT GAT GG-3′
TLR	5′-CAG GTG GAA TTG TAT CGC CT-3′	5′-CGA GGC TTT TCC ATC CAA TA-3′
HIF-1	5′-CGA AGT TAC AG CTT TCC GAC CAG-3′	5′-GTT TGT GTC GGT CAG CAC CAC T-3′
IL-10	5′-TTA ATA AGC TCC AAG ACC AAG G-3′	5′-GATO GATO GTA TGC TTC TAT GCA G-3′
Nfr2	5′-CTG AAC TCC TGG ACG GGA CTA-3′	5′-CGG TGG GTC TCC GTA AAT GG-3′

NF-κβ: Nuclear factor kappa B; COX-2: Cyclooxygenase; IL-6: Interleukin 6; TNF-α: Tumor necrosis factor-α; TLR: Toll-like receptors; HIF-1: Hypoxia-inducible factor 1-alpha; IL-10: Interleukin-10, Nfr2: Nuclear factor erythroid 2-related factor 2.

## Data Availability

All acquired data are systematically presented within the text. For additional inquiries, please direct them to the corresponding author.

## Supplementary Materials

The following supporting information can be downloaded at: https://www.mdpi.com/article/10.3390/molecules30102223/s1. Mass spectrometry analysis and High-performance liquid chromatography (HPLC) analyses of the peptide SGHPGAMGPVGPR, obtained from AminoTech (PDF).

## Abbreviations

IL-6	Intleukin-6
IL-10	Interleukin-10
TNF-alpha	Tumor Necrosis Factor-alpha
COX	Cyclooxygenase
TLR-4	Toll-like Receptor 4
NRF-2	Nuclear Factor Erythroid-2
HIF-1	Hypoxia-Inducible Factor 1
HO-1	Heme Oxygenase 1
NO	Nitric Oxide
SOD	Superoxide Dismutase
CAT	Catalase
GST	Glutathione S-Transferase
NF-κB	Nuclear Factor kappa
iNOS	Inducible nitric oxide synthase
LPS	Lipopolysaccharide
