# Supplementary material for: Effect of Synthetic Peptides Identified in the Bullfrog Skin on Inflammation and Oxidative Stress Control: An In Vitro Analysis
Source: Molecules. 2025 May 20;30(10):2223. doi: 10.3390/molecules30102223 (PMC12114019; doi:10.3390/molecules30102223)
Supplement: Supplementary file 1 [file molecules-30-02223-s001.zip › molecules-3595043-supplementary.pdf]

Mass spectrometry analysis

NH_2-Ser-Gly-His-Pro-Gly-Ala-Met-Gly-Pro-Val-Gly-Pro-Arg-COOH
(L:23112)

Model: LCMS-2020	Entrance Lens: +20,0 V	M	1219,3
RF Gain: 5000	Interface: ESI	[M+H]$^{+}$	1220,3
RF Offset: 5100	Nebulizing Gas Flow: 1,50 L/min	[M+2H]$^{+2}$	610,7
Mainrod Bias: +5,0 V	Drying Gas Flow: 15,00 L/min	[M+3H]$^{+3}$	407,4
Conversion Dynode: +10,0 kV	Interface Bias: -3,50 kV	[M+4H]$^{+4}$	305,8
Detector: -1,10 kV	Interface Bias: -3,50 kV	[M+5H]$^{+5}$	244,9
PG: 0,0e+000 Pa	Interface Current: 0,0 uA	[M+6H]$^{+6}$	204,2
IG: 0,0e+000 Pa	Heat Block Temp.: 150 C		
DL Temp.: 150 C			

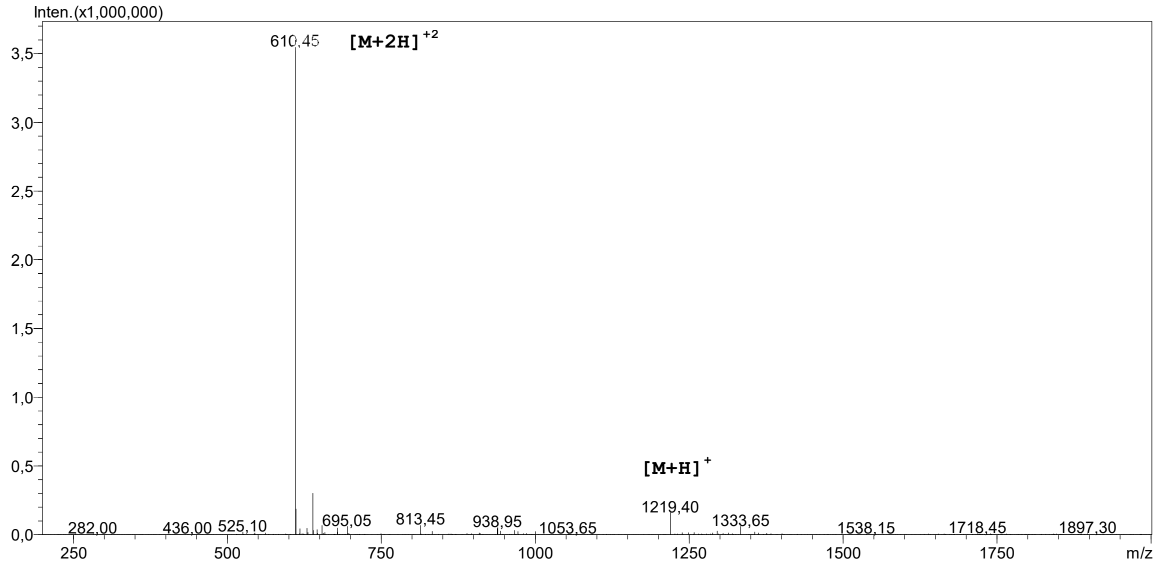

AMINOTECH PESQUISA E DESENVOLVIMENTO - HPLC ANALYSIS

C:\LabSolutions\Data\Project1\23112-LCF01.lcd

Acquired by	: System Administrator
Sample Name	: 23112-LCF
Sample ID	: 23112-LCF
Tray#	: 1
Vial#	: 1
Injection Volume	: 20
Data File	: C:\LabSolutions\Data\Project1\23112-LCF01.lcd
Method File	: C:\LabSolutions\Data\Project1\AmT-AnalCOLETA-ISO.lcm
Report Format File	: C:\LabSolutions\System\DEFAULT.lsr
Month-Day Acquired	: 14/11/2023
Month-Day Processed	: 14/11/2023

NH2-Ser-Gly-His-Pro-Gly-Ala-Met-Gly-Pro-Val-Gly-Pro-Arg-COOH

C:\LabSolutions\Data\Project1\23112-LCF01.lcd

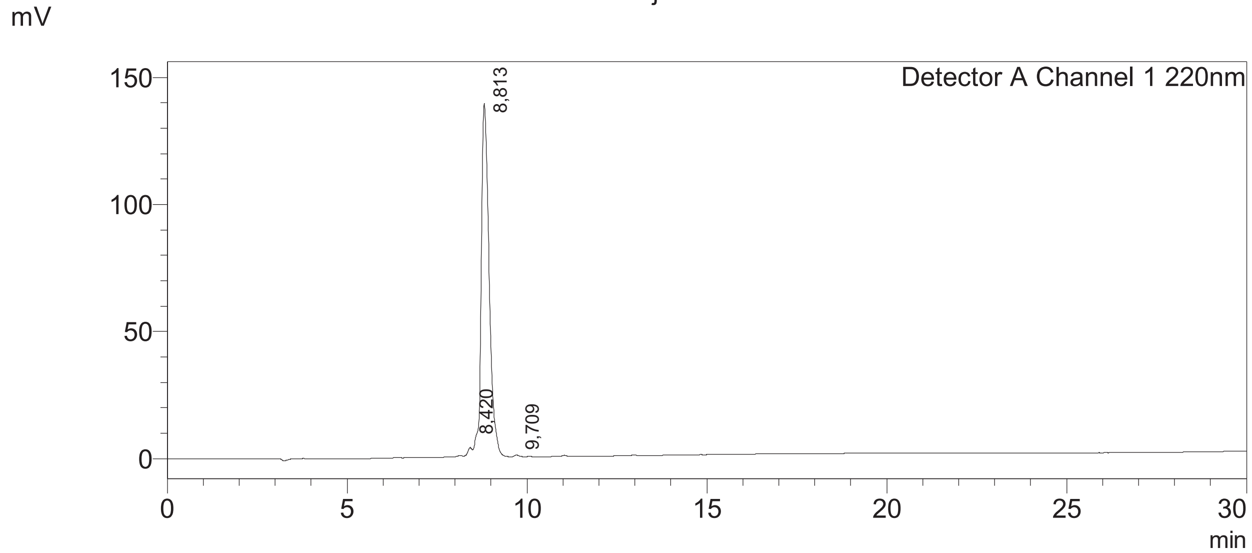

Peak Table

Detector A Channel 1 220nm

Peak#	Ret. Time	Area	Area%	Height	Height%	Conc.	Mark
1	8,420	45018	2,060	3807	2,647	2,060	M
2	8,813	2135185	97,682	139292	96,866	97,682	SV
3	9,709	5643	0,258	700	0,487	0,258	T
Total		2185847	100,000	143798	100,000		

Analysis conditions

ColumnRP-C18, 4,6x250mm - Flow : 1mL/min

Solvents : A=TFA 0,1%/H2O, B=CH3CN/H2O 9:1 com TFA 0,1%

Detector: 220nm - Gradient : 5 - 100% of B in 25 min.
